# Supplementary material for: Cx43 Hemichannel and Panx1 Channel Modulation by Gap19 and 10Panx1 Peptides
Source: Int J Mol Sci. 2023 Jul 18;24(14):11612. doi: 10.3390/ijms241411612 (PMC10380488; doi:10.3390/ijms241411612)
Supplement: Supplementary file 1 [file ijms-24-11612-s001.zip › ijms-2509008-supplementary.pdf]

Supplementary Materials: The following supporting information can be downloaded at: www.mdpi.com/xxx/s1, Figure S1: Enlarged display of representative current traces in Figure 1A. Figure S2: Flickering Panx1 channel closure activity induced by [10]Panx1 peptide. Figure S3: Enlarged display of representative current traces in Figure 3A.

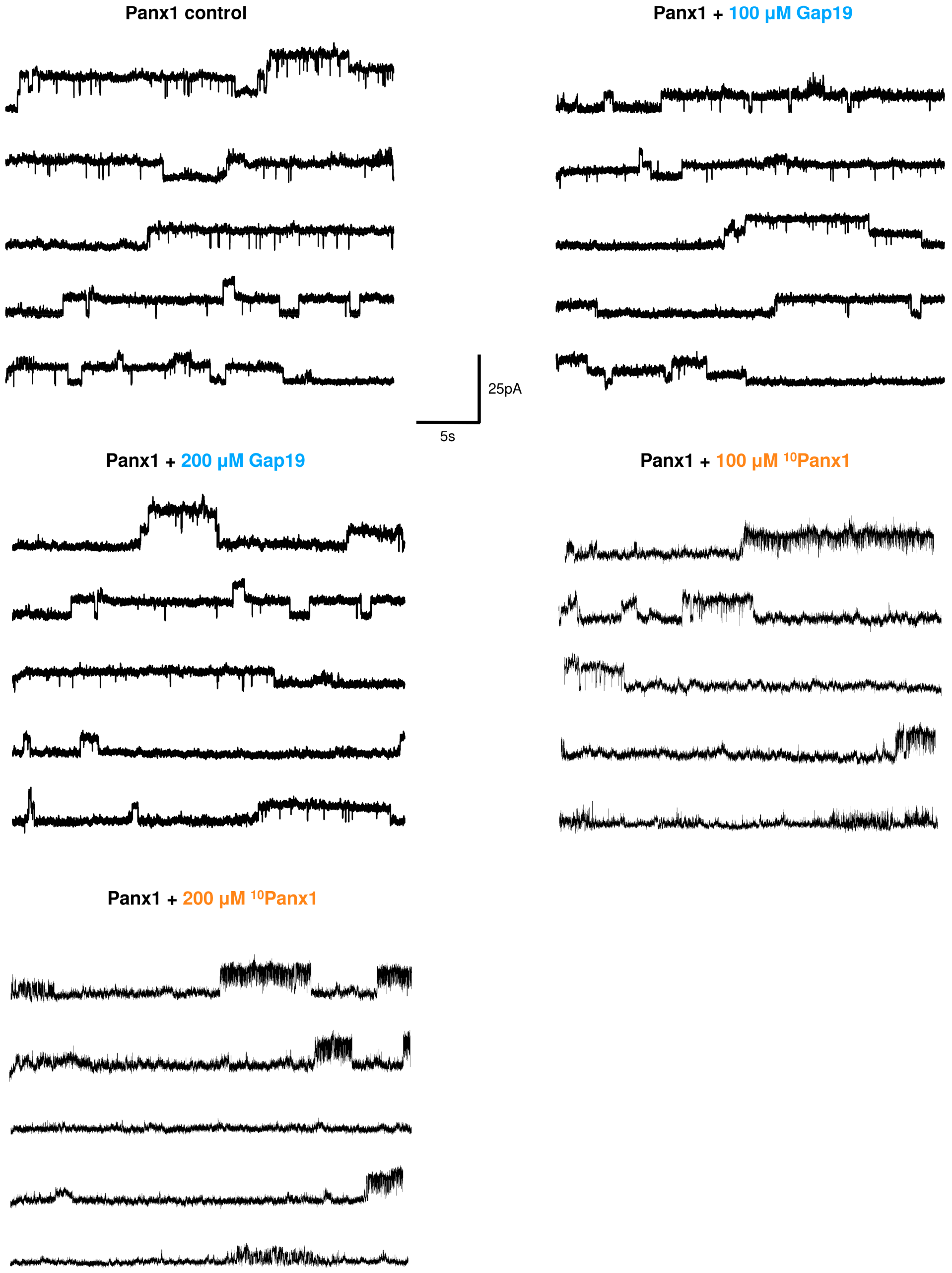

Figure S1: Enlarged display of representative current traces in Figure 1A.

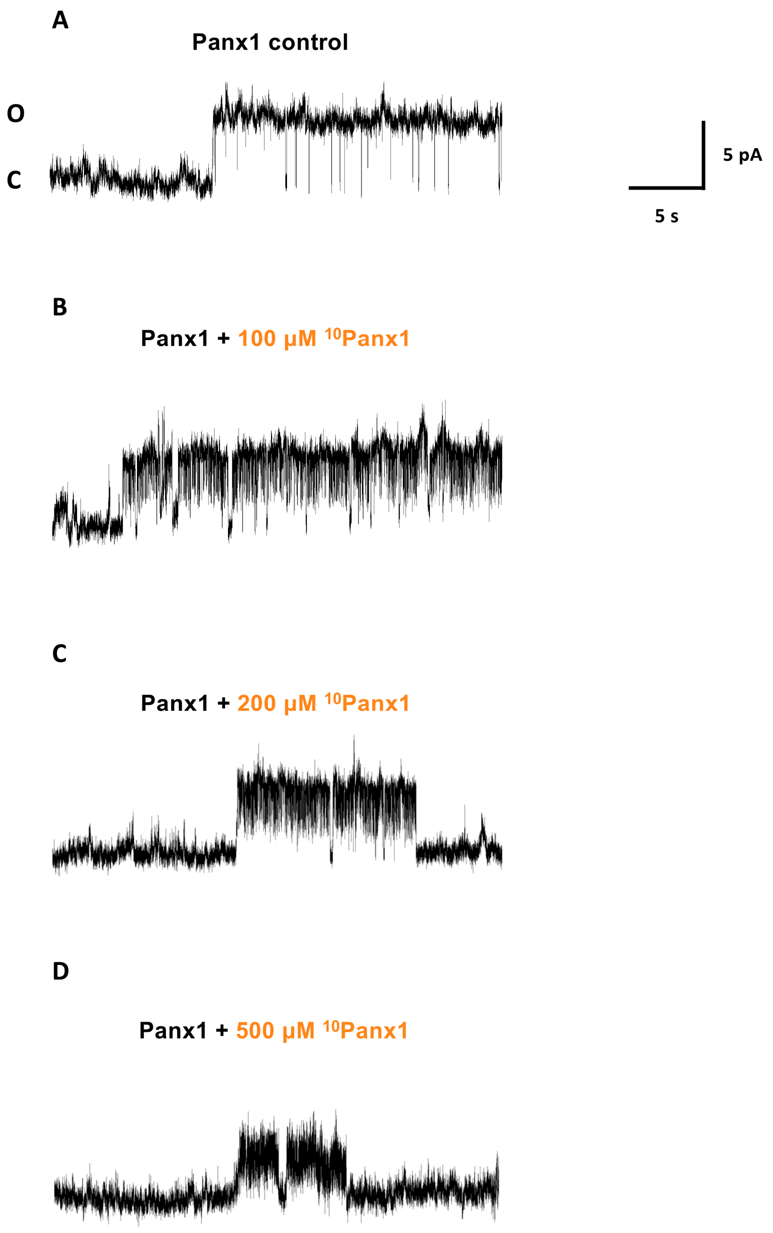

Figure S2: Flickering Panx1 channel closure activity induced by ^{10}Panx1 peptide. Single-channel recordings of Panx1 current activity illustrating flickering channel closure events in the presence of ^{10}Panx1; the flickering frequency increased with the ^{10}Panx1 concentration.

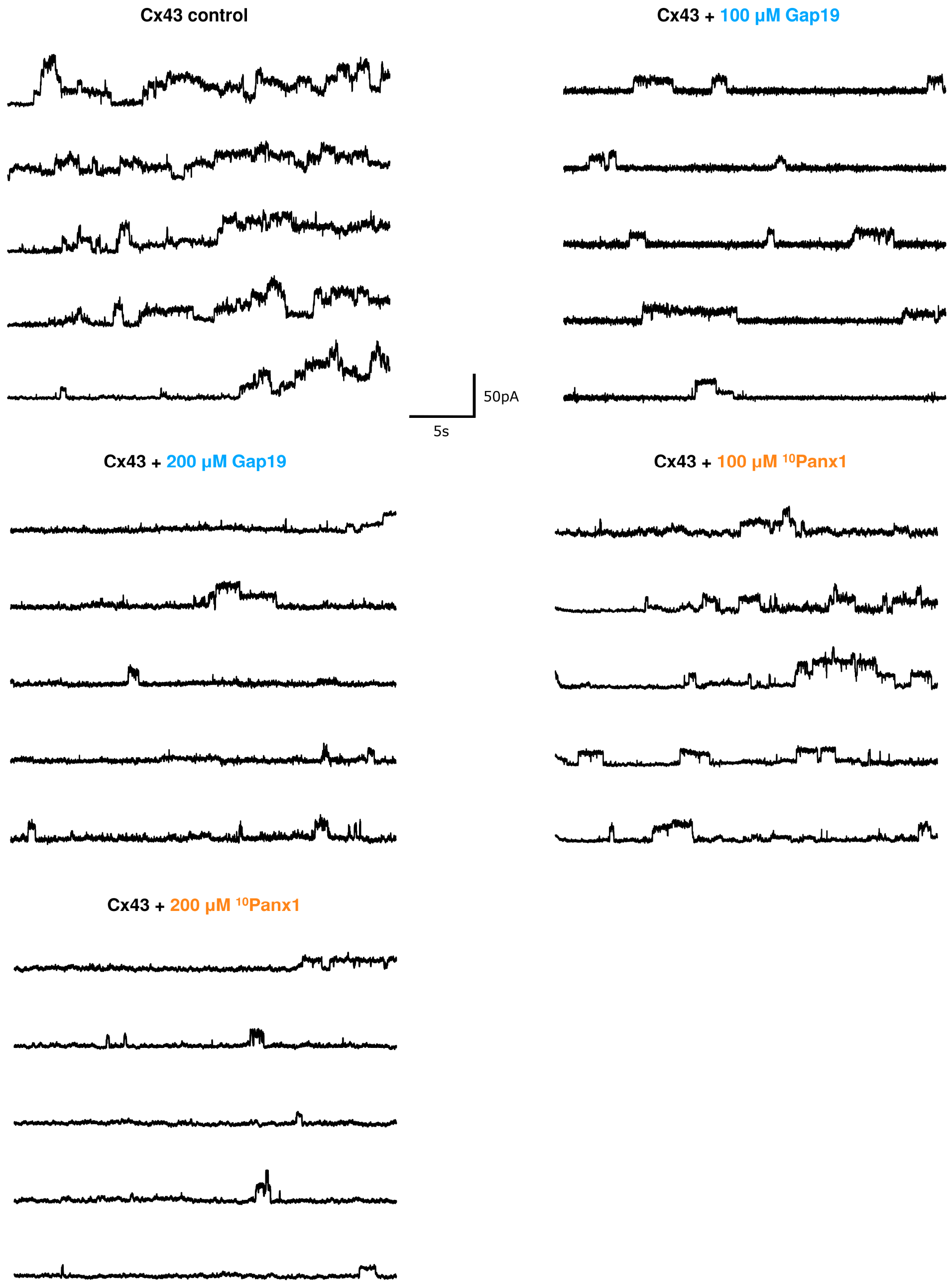

Figure S3: Enlarged display of representative current traces in Figure 3A.
